# A psychiatric medication, clozapine, induces autophagy and apoptosis in breast cancer cells through reactive oxygen species

**DOI:** 10.1371/journal.pone.0326224

**Published:** 2025-06-18

**Authors:** Ya-Chun Fan, Shih-Chao Lin, Po-Jung Lai, Pei-Chun Lai, Germain Maurus, Shiow-Yi Chen

**Affiliations:** 1 Department of Bioscience and Biotechnology, College of Life Sciences, National Taiwan Ocean University, Keelung, Taiwan; 2 Bachelor Degree Program in Marine Biotechnology, College of Life Sciences, National Taiwan Ocean University, Keelung, Taiwan; 3 Department of Aquaculture, College of Life Sciences, National Taiwan Ocean University, Keelung, Taiwan; University of Toronto, CANADA

## Abstract

Cancer patients with psychotic disorders have occasionally exhibited reduced tumor sizes following long-term antipsychotic treatment. Previous studies have shown that antipsychotic drugs, such as clozapine, could inhibit cancer cell proliferation, but the underlying mechanisms remain unclear. This study investigates the anti-tumor effects of clozapine on breast cancer cells and explores its mechanisms of action. We used clonogenic and MTT assays to assess cell proliferation, flow cytometry and western blotting analyses to evaluate cell cycle distribution, apoptosis, and autophagy following clozapine exposure. The results show that clozapine downregulates Cyclin D1, CDK4, and CDK6, while upregulating p21 and p27 in MCF-7 cells, leading to G0/G1 phase arrest. Clozapine exposure also increases reactive oxygen species (ROS), apoptosis and autophagy levels. Notably, treatment with the antioxidant α-Tocopherol restores cell viability and reduces ROS and autophagy, indicating that ROS plays a central role in clozapine-induced cytotoxicity. Additionally, inhibition of autophagy using chloroquine enhances clozapine-induced apoptosis and further reduces cell viability. These findings suggest that clozapine induces apoptosis and autophagy through ROS generation and that combining clozapine with autophagy inhibitors could sensitize MCF-7 cells to treatment. Furthermore, clozapine induces significant cytotoxicity in MDA-MB-231 cells, an aggressive, ER-negative breast cancer model, through similar ROS- and autophagy-mediated mechanisms. The addition of α-Tocopherol similarly rescued these cells from clozapine-induced cell death. Overall, our study demonstrates that clozapine suppresses the growth of both MCF-7 and MDA-MB-231 breast cancer cells by inducing cytotoxicity via ROS and autophagy, highlighting its potential as a therapeutic agent, especially in combination with autophagy inhibitors.

## Introduction

Breast cancer is the leading cause of cancer-related deaths among women globally, accounting for approximately 670,000 fatalities reported by the World Health Organization (WHO) in 2022 [1]. The statistics from the American Cancer Society indicate that over 2.3 million new cases were diagnosed worldwide in 2020. Additionally, the incidence of breast cancer has shown an upward trend, rising by 1% annually from 2012 to 2021, particularly among women under 50 and specific ethnic groups. Furthermore, by 2040, it is predicted that the global burden of breast cancer will exceed 3 million new cases and 1 million deaths annually due to population growth and aging [2,3]. Despite significant advancements in breast cancer treatment, the survival rates drastically diminish as the disease progresses to more advanced stages. According to the American Cancer Society, while the five-year survival rate for early-stage breast cancer exceeds 90%, this figure plummets to around 30% in later stages [2,3]. This stark contrast underscores the necessity for continued exploration of the underlying mechanisms driving breast cancer progression. Understanding these mechanisms is crucial for developing targeted therapies to improve survival rates, particularly in advanced cases. Breast cancer is a multifaceted disease, with its etiology influenced by a complex interplay of genetic, hormonal, environmental, nutritional, and lifestyle factors [4,5]. Consequently, there is an urgent need for more in-depth research to uncover how these factors contribute to breast cancer development and progression. Such investigations can pave the way for novel prevention strategies and more effective treatment options, including identifying new molecular subtypes, biomarkers, and therapeutic targets that could revolutionize clinical management.

Current breast cancer treatments primarily involve breast-conserving surgery, mastectomy, radiation therapy, and chemotherapy, with treatment regimens tailored to the patien’s age and cancer stage [6]. For patients with metastatic breast cancer, including aggressive triple-negative breast cancer (TNBC), chemotherapy remains the cornerstone of treatment, often combined with immunotherapy. Third-generation anthracycline-based regimens have been shown to extend survival in these patients. However, the associated risk of cardiac toxicity limits the use of anthracyclines as an adjuvant therapy, highlighting the need for alternative chemotherapeutic options [7].

Interestingly, antipsychotic medications, traditionally used to treat schizophrenia, have been associated with potential anticancer effects since at least 1955 [8]. Clinical studies have suggested that patients undergoing antipsychotic treatment for schizophrenia may have a lower incidence of certain cancers, including gastrointestinal and brain cancers [9,10]. *In vitro* studies have also demonstrated that clozapine, a second-generation antipsychotic, can inhibit cell proliferation through the induction of autophagic cell death in non-small cell lung carcinoma cells [11]. Given the high cost and time-intensive process of developing new anticancer drugs, there has been growing interest in repurposing existing medications, including antipsychotics, for cancer treatment [12]. Clozapine, known for its efficacy in treating refractory schizophrenia, has shown promise beyond its psychiatric applications [13]. It has been found to inhibit the maturation and differentiation of myeloid cells [14]. Moreover, studies have indicated that clozapine can significantly reduce tumor size and prolong the doubling time of breast cancer cells, specifically in the MDA-MB-231 cell line, in breast tumor-bearing mice [15]. Despite these findings, research on the relationship between clozapine and breast cancer remains limited. In this study, we aim further to investigate the interaction between clozapine and breast cancer cells, focusing on the underlying mechanisms by which clozapine exerts its anticancer effects. Our findings could provide new insights into the potential repurposing of clozapine as a therapeutic option in breast cancer treatment.

## Materials and methods

### Cell culture

Two breast cancer cell lines, MCF-7 and MDA-MB-231, were purchased from Bioresource Collection and Research Center (BCRC, Hsinchu, Taiwan) and cultured at 37^◦^C and 5% CO_2_ in RPMI-1640 and Leibovitz’s L-15 (L-15) media (Gibco, Billings, MT, USA) containing 10% of fetal bovine serum (FBS), respectively. When confluent, cells were washed twice with PBS and treated with 2 mL of TEG (0.025% of trypsin, 2.5 mM EDTA, and 2.8 mM glucose) for detachment. Cells were reseeded into a 10-cm petri dish in a ratio of 1:4–1:6 for subculture.

### Cell viability assays

Cells were planted in a 24-well plate overnight before adding different concentrations of clozapine (0, 12.5, 25, 37.5, and 50 μM) in fresh culture medium and incubated for 24, 48, and 72 hours. The control group was treated with 0.1% Dimethyl sulfoxide (DMSO) as a vehicle control. A 1 mg/mL of MTT stock solution was 10-fold diluted with PBS and added to each well (100 μg/mL, 100 μL/well). After incubation, the MTT solution was removed, and formazan was dissolved in isopropanol to quantify cell viability by measuring the optical density at 595 nm.

### Clonogenic assay

Cells were cultured in a 6-cm petri dish for 24 hours, washed with PBS, and cultured with clozapine-containing medium at different concentrations (0, 12.5, 25, 37.5, and 50 μM) for three additional days. The clozapine-containing medium was replaced with fresh medium without clozapine, and cells were cultured until the 14^th^ day. Cells were fixed in 10% formaldehyde for 30 minutes and stained with a 2% crystal violet solution. After removing excessive crystal violet solution and washing the wells with PBS, the distribution of colonies was recorded and quantified.

### Flow cytometry

Cells treated with or without clozapine for three days of incubation were collected using TEG buffer and washed with PBS. Cell pellets were resuspended with 300 μL of PBS. For cell cycle analysis, 1 mL of 99.5% alcohol was added to an eppendorf tube after centrifugation at 300 g for 5 minutes. The cells were then fixed at −20^◦^C for one hour, and the alcohol was removed. After removing alcohol, 10 mg/mL of RNase A and propidium iodide (PI) dissolved in PBS were used to digest RNA and stain DNA, followed by incubation for 30 minutes in the dark. For ROS measurement, cells were incubated with a medium containing H_2_DCF-DA fluorescent reagent for 30 min at 37^◦^C in the dark, trypsinized with TEG buffer, and collected to Eppendorf prior to centrifugate at 300 g for 5 minutes. Cells were analyzed with fluorescent intensity to quantify ROS levels.

For autophagy or apoptosis analysis, cells were treated with clozapine and collected as in previous procedures, followed by the addition of 0.25 μg/mL of acridine orange fluorescent dye or apoptotic reagents provided by Dead Cell Apoptosis Kits with Annexin V (Invitrogen, Waltham, MA, USA) before analyzing with flow cytometry.

### TUNEL assay

Cells were cultured in 24-well plates containing cover glasses at a density of 2 × 10^4^ cells/well for 24 hours. The medium was replaced by a medium containing clozapine (50 μM) and incubated for 72 hours. According to the manufacturer’s protocol, the apoptotic cells were stained with Click-iT™ Plus TUNEL Assay Kits (Invitrogen). Cells on the cover glasses were then examined by inverted fluorescent microscopy.

### Western blot

Cell lysates were collected with the lysis buffer supplemented with 1X phosphatase inhibitor, 1X protease inhibitor, and ProteoJET^TM^. The mixture was boiled at 95^◦^C for 10 min following the quantification of BCA assay to quantify each sample concentration by ELISA reader at OD_595 nm_. Protein samples were separated by molecular weights in SDS-PAGE and transferred to a PVDF membrane. The membrane was then blocked by 5% non-fat milk in PBS with 0.1% Tween-20, followed by hybridizing primary antibodies in a 1:1000 diluted ratio overnight at 4^◦^C. The next day, the membrane was washed three times with PBST and hybridized with 1:10000 secondary antibodies for one hour at room temperature. All primary antibodies used in this study were purchased from Cell Signaling^®^, including p21/Cip1 (#2947), p27/Kip1 (#3686), Cyclin D1 (#2978), CDK4 (#2906), CDK6 (#3136), LC-3B (#2775), Beclin-1 (#3495), Ayg7 (#2631), Atg5 (#2630), cleaved PARP (cPARP) (#9541), cleaved caspase 9 (#9501) except β-actin (Sigma-Aldrich^®^, #A5441). After hybridization, the membrane was washed three times with PBST and was added with enhanced chemiluminescence (ECL) to react with the peroxidase on secondary antibodies. Luminescent images were captured on film in the dark. The intensity of each band on the film was further quantified using ImageJ software.

### Statistical analysis

All data were compared and evaluated for statistically significant differences using GraphPad Prism v10.0 software. Student’s unpaired t-test was primarily used to determine significant differences between groups. An α-value of 0.05 was used as the threshold for rejecting the null hypothesis. The data are presented as mean ± standard deviation (S.D.). No multiple testing correction was applied.

## Results

### Clozapine inhibited MCF-7 cell proliferation and colony formation

**Fig 1:**
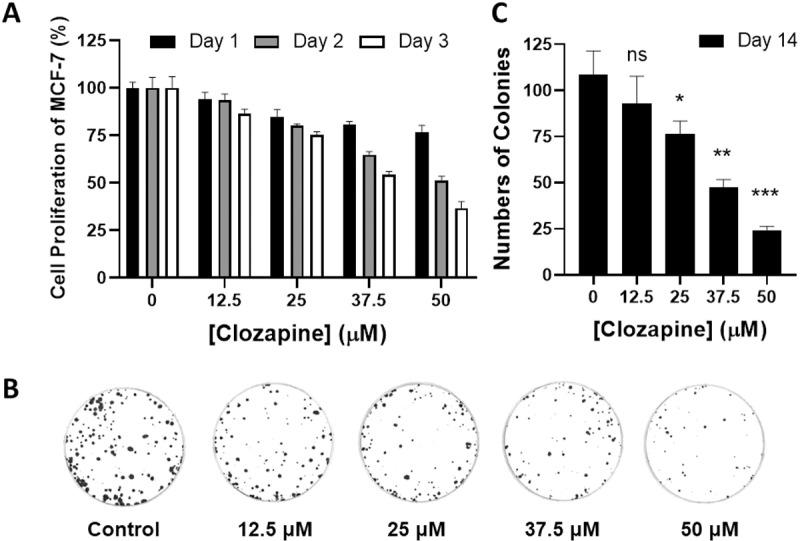
Clozapine inhibited cancerous growth of MCF-7 cells. (A) MCF-7 cells were seeded into 24-well plates at a density of 2 × 104 cells/well for 24h, followed by incubating with the indicated concentration of clozapine for 1 to 3 days. Cell proliferation was then measured using an MTT assay. (B) 500 cells were seeded in a 60 mm Petri dish and incubated with clozapine at various concentrations for 14 days. Representative images of colony formation assays were shown. (C) Colonies were stained with crystal violet and quantified. Data were presented as mean ± standard deviation (S.D.) P value below 0.05 was denoted with the asterisks, where * indicates < 0.05; ** means < 0.01; *** signifies < 0.001.

To assess the antiproliferative effects of clozapine on breast cancer cells, we first utilized MTT and clonogenic assays on the MCF-7 cell line. MCF-7 cells were exposed to varying concentrations of clozapine (0, 12.5, 25, 37.5, and 50 μM) over three days. The results from the MTT assay demonstrated a dose-dependent inhibition of cell proliferation by clozapine. Specifically, as the concentration of clozapine increased, there was a corresponding decrease in the proliferation rates of MCF-7 cells, with the inhibitory effect becoming more pronounced over time (Fig 1A).

Additionally, the clonogenic assay was employed to evaluate the long-term effects of clozapine on the colony-forming ability of MCF-7 cells. Our result showed a substantial reduction in colony formation (Fig 1B). Notably, concentrations of clozapine above 25 μM significantly decreased the number of colonies, further supporting the conclusion that clozapine exhibited strong cytotoxic effects on MCF-7 cells (Fig 1B and 1C). These findings collectively indicate that clozapine impeded the proliferation of MCF-7 cells and markedly reduced their ability to form colonies.

### Clozapine induced cell cycle arrest of MCF-7 cells at G0/G1

**Fig 2:**
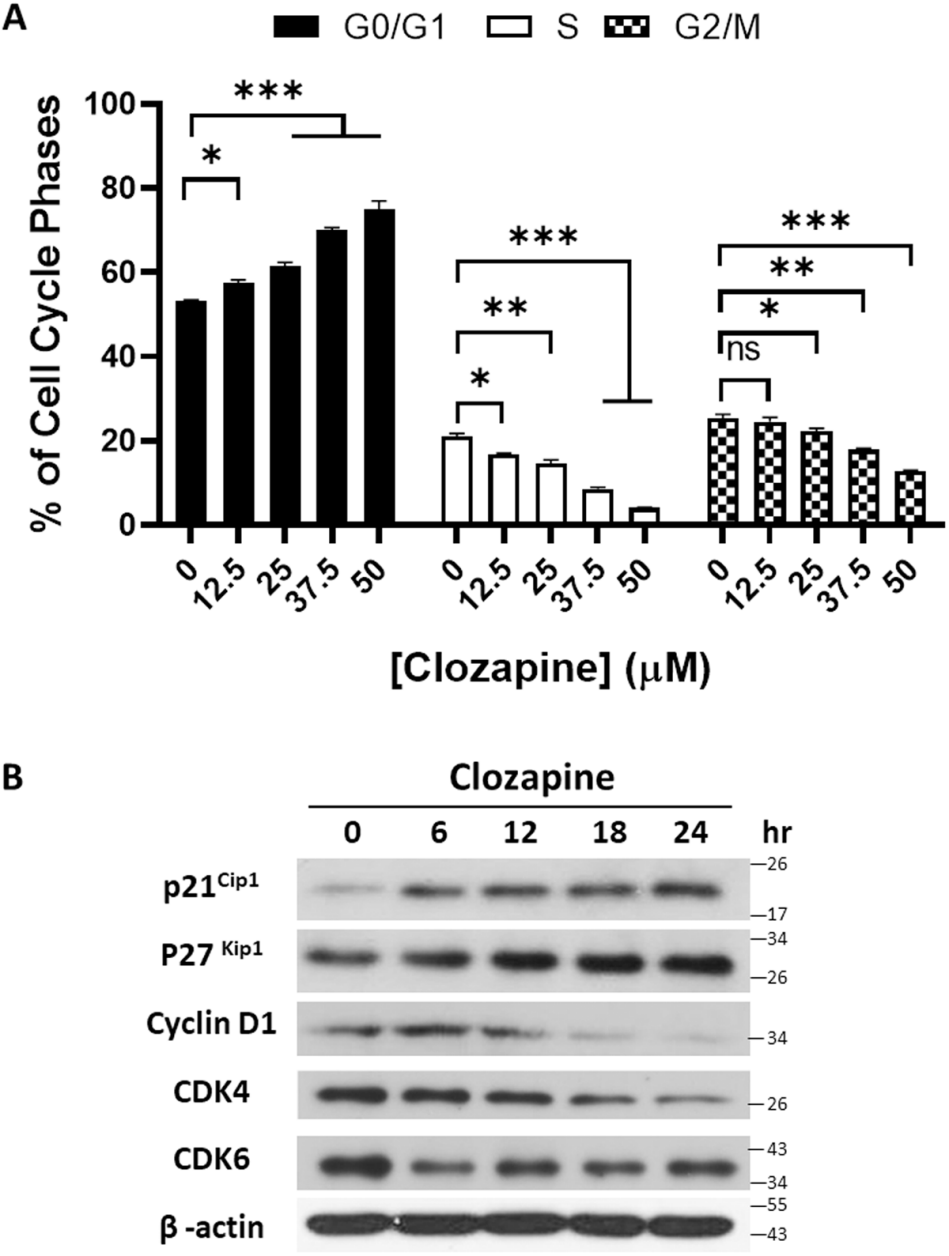
Clozapine arrested cell cycle of MCF-7 cell. (A) MCF-7 cells were treated with the indicated concentration of clozapine for 72 h, harvested, stained with propidium iodide, and analyzed using a flow cytometer. Data were presented as mean ± standard deviation (S.D.) (B) MCF-7 cells were treated with 50 μM clozapine for 6, 12, 18, and 24h, after which total protein lysates were collected and analyzed by western blotting using antibodies against cell-cycle-related proteins. β-actin was used as an internal control to confirm equal protein loading. P value below 0.05 was designated with the asterisks, where * indicates < 0.05; ** means < 0.01; *** signifies < 0.001.

To explore the mechanisms by which clozapine inhibits MCF-7 cell growth, we investigated its effects on cell cycle progression. Given that cell death and cell cycle arrest are two primary mechanisms through which anticancer agents exert their effects [16,17], we focused on analyzing the impact of clozapine on the cell cycle. MCF-7 cells were treated with varying concentrations of clozapine for three days, followed by DNA content analysis using propidium iodide (PI) staining and flow cytometry. The results revealed a significant dose-dependent increase in the proportion of MCF-7 cells arrested in the G0/G1 phase (Fig 2A).

To further understand the molecular basis of this cell cycle arrest, we examined the expression levels of key proteins involved in cell cycle regulation. Western blot analysis showed that clozapine treatment led to a marked reduction in CDK4 and CDK6 levels, both of which are critical for G1 phase progression. Additionally, the expression of Cyclin D1, a protein typically overexpressed in breast cancer cells [18,19], was significantly decreased following clozapine treatment, particularly after 18 hours of exposure. In contrast, the cyclin-dependent kinase inhibitors p21 and p27 levels were elevated in clozapine-treated MCF-7 cells, indicating that clozapine may induce G0/G1 arrest by upregulating these inhibitors (Fig 2B).

These findings suggest that clozapine not only exerted cytotoxic effects on MCF-7 cells but also induced cell cycle arrest at the G0/G1 phase, thereby inhibiting cell proliferation. This dual action of clozapine underscores its potential as a therapeutic agent in breast cancer treatment by targeting both cell growth and survival pathways.

### Induction of autophagy and apoptosis in clozapine-treated MCF-7 cells

**Fig 3:**
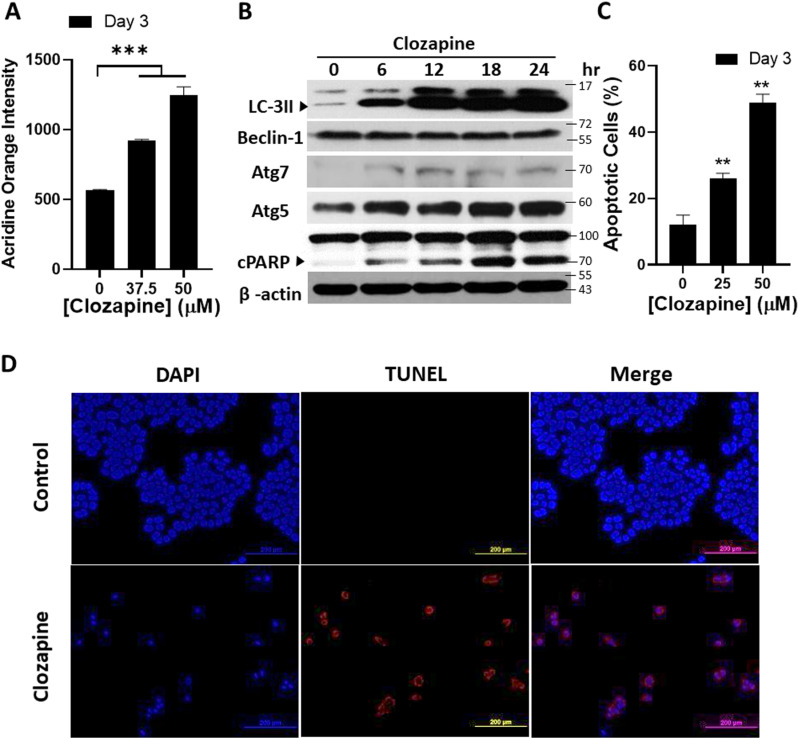
Clozapine modulated apoptosis and autophagy in MCF-7 cells. MCF-7 cells were seeded into 6-well plates at a density of 10^5^ cells/well and treated with an indicated concentration of clozapine for 72 h. Cells were harvested and stained with (A) 0.25 μg/mL of acridine orange and (C) fluorescein-conjugated Annexin V antibody and propidium iodide for flow cytometric analysis of autophagy and apoptosis, respectively. (B) The protein levels of autophagic and apoptotic modulators and mediators, with or without 50 μM of clozapine treatment, were measured by western blotting. (D) The fragmented DNA resulting from apoptosis was fluorescently imaged using a TUNEL assay. Results were presented as mean ± standard deviation (S.D.) ** means < 0.01; *** signifies < 0.001.

Given our previous findings that clozapine induces cell death in non-small cell lung cancer cells through autophagy [11], we sought to determine whether a similar mechanism might be at play in MCF-7 breast cancer cells. To investigate this, we first assessed the presence of acidic vesicular organelles (AVOs), indicative of autophagy, in clozapine-treated cells. MCF-7 cells were treated with clozapine for three days, followed by staining with acridine orange, a fluorophore that accumulates in AVOs. Flow cytometry analysis revealed a significant increase in AVO-positive cells, with the extent of AVO accumulation correlating with higher clozapine concentrations (Fig 3A). This suggests that clozapine activates the autophagic pathway in MCF-7 cells.

To further confirm the induction of autophagy, we examined the expression levels of autophagy-related proteins. Western blot analysis showed that clozapine treatment led to a marked increase in the expression of critical autophagic markers [20,21], including Atg7, Atg12/5, and LC3-II, as well as Beclin-1 (Fig 3B). These results strongly indicate autophagy is activated in MCF-7 cells following clozapine exposure.

In addition to autophagy, apoptosis is another common pathway through which cancer cells undergo programmed cell death [22]. To explore whether clozapine also induces apoptosis in MCF-7 cells, we performed flow cytometry analysis using Annexin V/ PI staining, which detects phosphatidylserine externalization, a hallmark of early apoptosis. The results showed a dose-dependent increase in the percentage of apoptotic cells, with approximately 50% of the cells undergoing apoptosis after three days of treatment with 50 μM clozapine (Fig 3C). This finding was further supported by the TUNEL assay, which revealed increased DNA fragmentation, a characteristic of apoptosis, in clozapine-treated cells compared to the control group (Fig 3D).

Moreover, we observed a significant increase in the expression of cleaved PARP-1 (cPARP-1), a marker of apoptosis, in clozapine-treated cells [23]. The concurrent increase in LC3-II expression suggests that both autophagy and apoptosis are involved in the cytotoxic effects of clozapine on MCF-7 cells (Fig 3B). Together, these findings indicate that clozapine exerts its cytotoxic effects on MCF-7 cells through the induction of both autophagy and apoptosis, highlighting its potential as a multifaceted therapeutic agent in breast cancer treatment.

### Clozapine inhibited cell proliferation of MCF-7 via induction of reactive oxygen species

**Fig 4:**
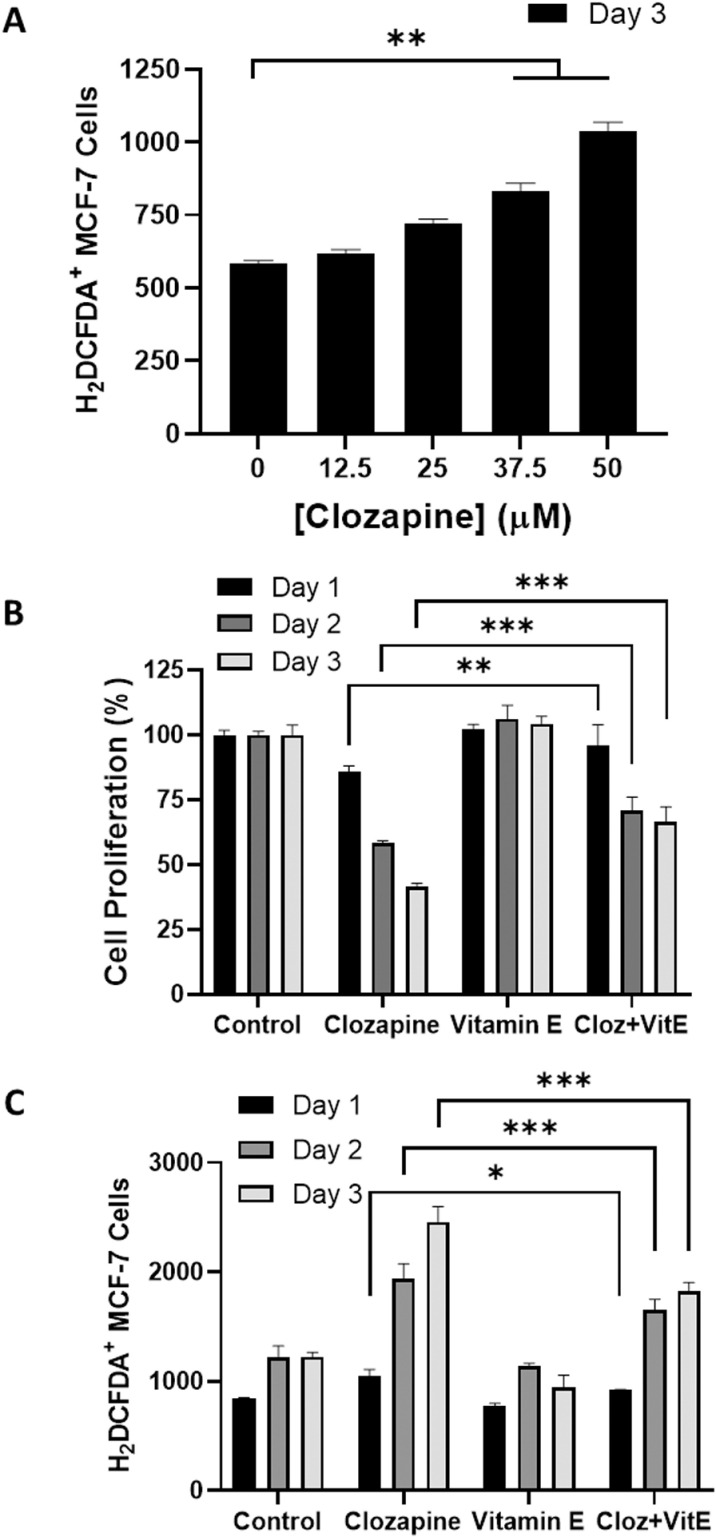
Clozapine suppressed MCF-7 cell growth via modulation of ROS. (A) MCF-7 cells were incubated with the indicated concentration of clozapine for up to 72 h and labeled with H_2_DCFDA (10 μM) to measure the total intracellular ROS content by flow cytometry. (B and C) Cells were treated with or without vitamin E (α-Tocopherol) to determine. (B) cell proliferation by MTT assay or (C) intracellular ROS content by flow cytometry. Cells without treatment were assigned 100% viability to emphasize the proliferation changes following clozapine treatment. Results were presented as mean ± standard deviation (S.D.). P value below 0.05 was denoted with the asterisks, where * indicates < 0.05; ** means < 0.01; *** signifies < 0.001.

Reactive oxygen species (ROS) are crucial in cellular signaling and homeostasis, acting as secondary messengers in various signaling pathways [24,25]. However, elevated ROS levels can lead to oxidative stress, which is detrimental to cancer cells [26,27]. To investigate whether ROS induction is a mechanism through which clozapine exerts its cytotoxic effects on MCF-7 cells, we measured intracellular ROS levels using the fluorescent probe H_2_DCFDA, followed by flow cytometry analysis. The results indicated a dose-dependent increase in ROS levels in clozapine-treated MCF-7 cells (Fig 4A). To further confirm the role of ROS in clozapine-induced cytotoxicity, we treated the cells with α-Tocopherol, a potent antioxidant, and isoform of vitamin E, to counteract the ROS generated by clozapine. The addition of α-Tocopherol significantly reduced the cytotoxic effects of clozapine, as evidenced by a 25% increase in cell viability compared to cells treated with clozapine alone (Fig 4B). This protective effect was accompanied by a corresponding decrease in ROS levels (Fig 4C).

**Fig 5:**
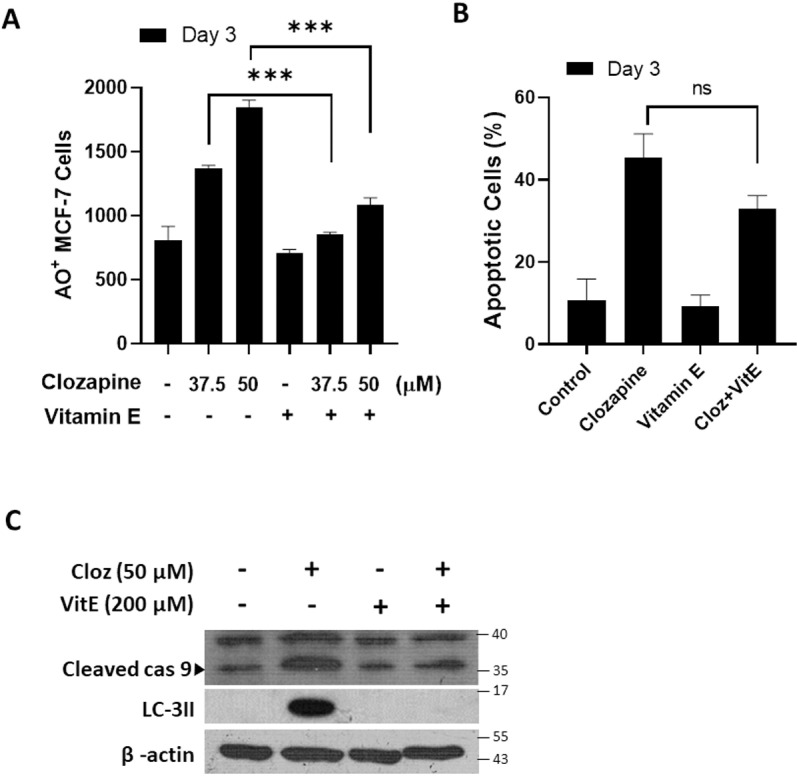
Clozapine enhanced apoptosis and autophagy of MCF-7 cells via modulation of ROS. MCF-7 cells were incubated with clozapine for 72 h, with or without treatment with vitamin E, and then (A) stained with acridine orange to identify autophagic cells or (B) with fluorescein-conjugated Annexin V antibody and propidium iodide to identify apoptotic cells, followed by flow cytometry analysis. (C) Autophagy- and apoptosis-associated proteins in clozapine-treated MCF-7 cells were collected and measured by western blotting. Data were plotted as mean ± standard deviation (S.D.). P value below 0.05 was denoted with the asterisks, where * indicates < 0.05; ** means < 0.01; *** signifies < 0.001.

Given that ROS are known to mediate both apoptosis and autophagy [28], we further assessed the impact of α-Tocopherol on these pathways in clozapine-treated MCF-7 cells. Flow cytometry analysis showed a substantial reduction in the percentages of both autophagic (Fig 5A) and apoptotic (Fig 5B) cells following α-Tocopherol treatment. Additionally, the levels of cleaved caspase-9 and LC3-II proteins, markers of apoptosis and autophagy, respectively, were significantly reduced (Fig 5C). These findings strongly suggest that ROS is a major mediator of clozapine-induced apoptosis and autophagy, thereby contributing to the overall cytotoxicity of clozapine in MCF-7 breast cancer cells.

### Chloroquine enhanced cytotoxicity effect of clozapine in MCF-7 cells

**Fig 6:**
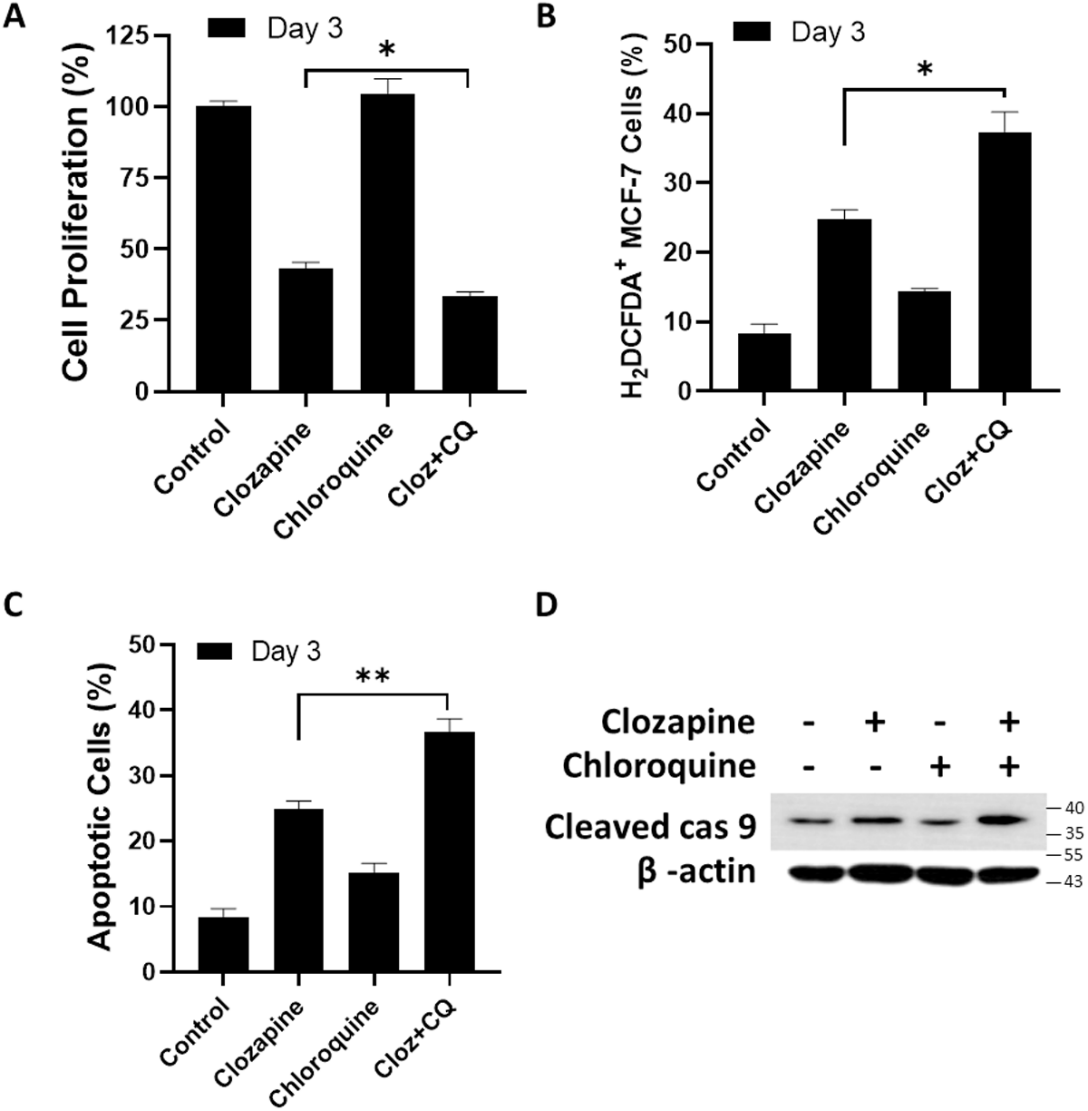
Chloroquine boosted apoptosis induced by clozapine. MCF-7 cells were treated with 50 μM of clozapine for 72 h, with or without chloroquine, followed by measuring (A) cell proliferation via MTT assay, (B) intracellular ROS content by H_2_DCFDA uptake assay, and (C) apoptotic percentages by flow cytometry. (D) Total protein lysates from each treatment of clozapine and/or chloroquine were collected to assess cleaved caspase 9 levels after 3 days of incubation. Data were plotted as mean ± standard deviation (S.D.). P value below 0.05 was denoted with the asterisks, where * indicates < 0.05; ** means < 0.01.

To further elucidate the role of autophagy in clozapine-induced cell death, we investigated the effects of combining clozapine with chloroquine, an inhibitor of autophagosome-lysosome fusion. MCF-7 cells were co-treated with clozapine and chloroquine, and their proliferation and intracellular ROS levels were assessed using MTT assays and H_2_DCFDA-based flow cytometry, respectively. Surprisingly, the co-treatment of MCF-7 cells with clozapine and chloroquine resulted in a more pronounced suppression of cell growth compared to clozapine treatment alone (Fig 6A). This enhanced cytotoxicity was accompanied by a significant increase in intracellular ROS levels in the co-treated cells (Fig 6B). Our analysis further revealed that the combination treatment led to an increased percentage of apoptotic cells and elevated levels of cleaved caspase-9, a key mediator of apoptosis (Fig 6C and 6D). This indicates that the inhibition of autophagy by chloroquine may shift the balance towards apoptosis, thereby potentiating the cytotoxic effect of clozapine in MCF-7 cells. These findings suggest that while autophagy may initially act as a survival mechanism in response to clozapine-induced stress, its inhibition by chloroquine forces the cells into apoptosis, leading to enhanced cell death.

### Clozapine induced cell death in MDA-MB-231 cells

**Fig 7:**
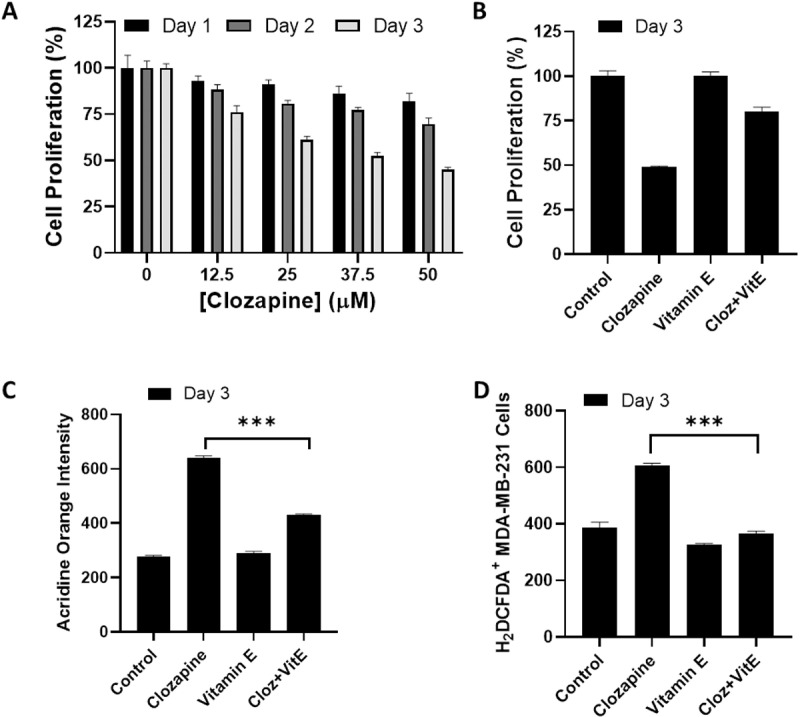
Clozapine exhibited cytotoxicity against MDA-MB-231 cells. (A) MDA-MB-231 cells were treated with 50 μM of clozapine for the indicated concentration and time points, up to 72 h, followed by an MTT assay to determine the cell proliferation. (B-D) α-Tocopherol (vitamin E) was used to counteract ROS triggered by clozapine after 3 days of incubation, followed by (B) cell proliferation assessed by MTT assay, (C) autophagic cells, and (D) intracellular ROS content, with the latter two assays were conducted by flow cytometry. Data were presented as mean ± standard deviation (S.D.). P value below 0.05 was denoted with the asterisks, where *** means < 0.001.

Finally, to determine whether the cytotoxic effects of clozapine are specific to MCF-7 cells or extend to other breast cancer cell lines, we investigated its impact on MDA-MB-231 cells, a more aggressive and invasive breast cancer cell line lacking estrogen receptors (ER-negative) [29]. Using the same experimental conditions as in the MCF-7 studies, we treated MDA-MB-231 cells with clozapine. The results showed that clozapine induced significant cytotoxicity in MDA-MB-231 cells, similar to its effects on MCF-7 cells (Fig 7A). ROS also appeared to be the primary mediator of clozapine-induced cell death, as α-Tocopherol significantly rescued clozapine-treated MDA-MB-231 cells (Fig 7B). Furthermore, the addition of α-Tocopherol, a potent antioxidant, could mitigate clozapine-induced cytotoxicity. The decreased AO^+^ and H_2_DCFDA^+^ cells following α-Tocopherol treatments indicated that α-Tocopherol effectively reduced ROS levels and partially rescued the cells from clozapine-induced death (Fig 7C and 7D). These findings suggest that, similar to MCF-7 cells, ROS is a primary mediator of clozapine-induced cell death in MDA-MB-231 cells.

## Discussion

The findings from this study extend our understanding of clozapine’s anticancer properties, building on previous research that demonstrated its efficacy in various cancer cell lines, including non-small cell lung cancer and breast cancer. Our results reveal that clozapine inhibited cell proliferation in both MCF-7 and MDA-MB-231 breast cancer cells, with ROS identified as a key mediator of this effect. Additionally, inhibiting ROS reduced autophagy and rescued cell survival, demonstrating a consistent mechanism in both cell lines. These results highlight clozapine’s potential as a broad-spectrum therapeutic agent in breast cancer treatment, capable of inducing cytotoxicity through ROS and autophagy pathways.

Clinically, diagnosing whether specific breast cancer is ER-positive or negative is crucial, as the testing results guide the treatment decisions made by physicians [30]. MDA-MB-231 cells, which lack estrogen receptors (ER-negative) but exhibit active and constitutive expression of Rho GTPase [31], are regarded as a more aggressive subtype. Nonetheless, both breast cancer cell types responded similarly to clozapine treatment (Fig 5 and 7), indicating that clozapine’s anticancer effects extend beyond specific breast cancer subtypes. This broad activity suggests clozapine’s therapeutic potential in treating diverse forms of breast cancer.

While we primarily focused on the ROS-mediated mechanisms exerted by clozapine, the upstream mechanism leading to ROS accumulation remains an important topic for further investigation. Numerous studies suggest that mitochondrial dysfunction is a key driver of ROS generation [32–34]. A recent study reported that clozapine induced cell death in pheochromocytoma cells through mitochondrial membrane potential (MMP) collapse, thereby, causing ROS accumulation [35]. Given the critical role of mitochondria in cancer cell metabolism, future studies should explore clozapine’s effects on mitochondrial respiration and metabolic enzyme activity in breast cancer cells to further elucidate its mechanism of action.

Beyond ROS-mediated effects, other pathways contributing to clozapine’s anticancer activity also warrant further exploration. Both first-generation (FGA) and second-generation (SGA) antipsychotics target dopamine receptors [36], and increasing evidence suggests that dopamine receptor signaling is implicated in breast cancer development [37,38]. The downregulation of dopamine receptor expression has been shown to suppress proliferation and enhance apoptosis in breast cancer cells, supporting the idea that clozapine may exert an additional anticancer effect by blocking dopamine receptors, thereby contributing to its observed cytotoxicity [39,40].

Notably, our experiment in Fig 6 showed that the reduction of autophagy by chloroquine enhanced the cytotoxic effect of clozapine against breast cancer cells. Initially, we expected to see a decrease in the cytotoxicity of clozapine following the suppression of autophagy, based on clozapine-induced autophagic cell death in non-small cell lung carcinoma cells [11]. However, the converse results provided us with a new insight into considering the role of autophagy during antipsychotic therapy. Autophagy triggered by clozapine seemed to be a responsive mechanism promoting cell survival rather than facilitating the overall cytotoxicity of clozapine [41]. Reviewing previous literature makes it much more apparent that there is close crosstalk between apoptosis and autophagy, and suppressing one of the cell death pathways could further activate the other [42,43]. As a result, the inhibition of autophagy to speed up the cell death process in breast cancers could be taken into account as a combinational therapy when drug resistance becomes a concern.

Despite the evidence presented in this study, which shows that clozapine suppressed breast cancer proliferation and is supported by previous studies, some discrepancies remain. Some epidemiological studies suggested antipsychotic therapy may increase breast cancer risk by elevating prolactin concentration [44,45]. However, clozapine, one of the atypical SGAs, barely induces prolactin; less than 5% of clozapine-received patients exhibited an increase in prolactin [46]. More recently and conclusively, a more extensive population-based cohort study of more than 150,000 patients examining the association of breast cancer risk and psychotropic medication use indicated no association between invasive breast cancer risk and antipsychotics, regardless of typical, atypical antipsychotics or lithium remedy. Also, there is no association between in situ breast cancer risk and atypical antipsychotic medication in this sizeable epidemiologic study [47].

This study suggests that clozapine exerts anticancer effects in MCF-7 and MDA-MB-231 breast cancer cells, potentially through ROS-induced apoptosis and autophagy mechanisms. Although more research is needed to fully understand the implications of these findings, particularly in clinical settings, our results indicate that clozapine holds significant promise as a potential therapeutic agent in the fight against breast cancer. In this research, we focused on investigating the anti-cancer properties of clozapine due to its unique pharmacological profile and clinical significance. Clozapine is widely regarded as the most effective antipsychotic for treatment-resistant schizophrenia and schizoaffective disorder, particularly for patients at increased risk of suicidal behavior. These therapeutic advantages make clozapine an ideal candidate for exploring its potential anti-cancer effects. Unlike other antipsychotics, clozapine has distinct mechanisms of action, which led us to hypothesize that it might also exhibit unique anti-tumor properties. Therefore, we concentrated on clozapine in this investigation to explore its effects on breast cancer cells. Expanding future research to include other antipsychotic drugs may help to compare their potential anticancer effects further and broaden the scope of our findings.

We also concede that the study was limited to *in vitro* 2D models, which may not fully capture the complexity of *in vivo* tumor dynamics. While our use of 2D models, breast cancer cells, allowed us to perform detailed mechanistic studies on cell cycle regulation, apoptosis, and autophagy in a controlled environment, we recognize that 3D models or spheroid cultures could provide a more accurate representation of the *in vivo* tumor microenvironment. These 3D models offer enhanced physiological relevance by mimicking the architectural and cellular heterogeneity of tumors, which can lead to more meaningful insights into drug efficacy. We understand that *in vivo* studies are essential for validating the therapeutic potential of clozapine in a more complex biological system. Future work will also expand our research by incorporating 3D spheroid and *in vivo* models to better assess clozapine’s anticancer effects in settings that more closely mimic human tumor biology. These approaches will enable us to evaluate clozapine’s therapeutic potential more comprehensively and in greater detail.

## Supporting information

S1 File Raw images. Uncropped western blog images used in the current study. (PDF)

S2 File Minimal data set. Uncropped western blog images used in the current study. (RAR)
