# Supplementary material for: A psychiatric medication, clozapine, induces autophagy and apoptosis in breast cancer cells through reactive oxygen species
Source: PLoS One. 2025 Jun 18;20(6):e0326224. doi: 10.1371/journal.pone.0326224 (PMC12176217; doi:10.1371/journal.pone.0326224)
Supplement: S1 File — (PDF) [file pone.0326224.s001.pdf]

Fig2B LC3II

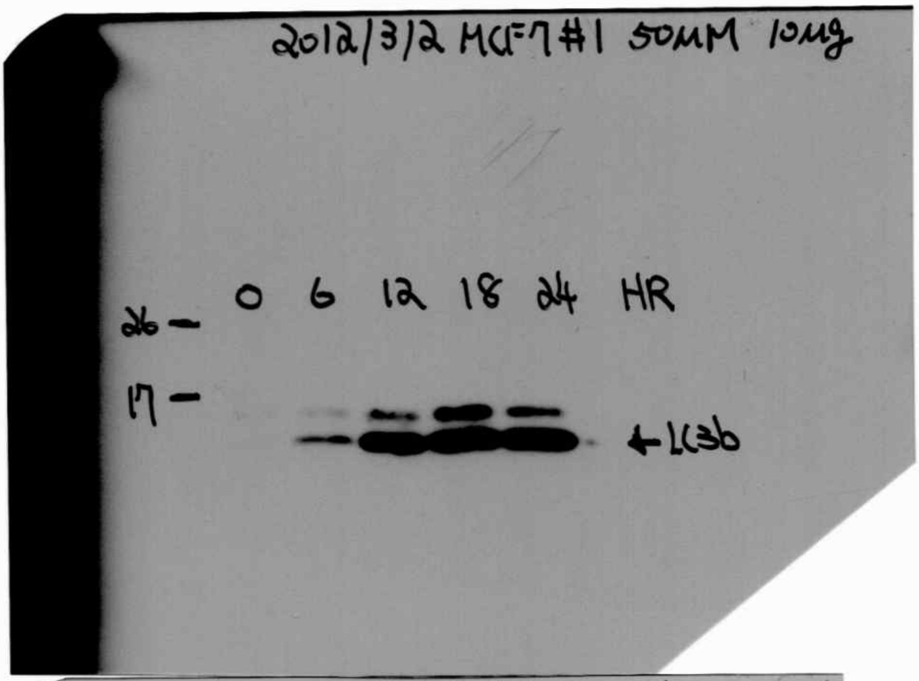

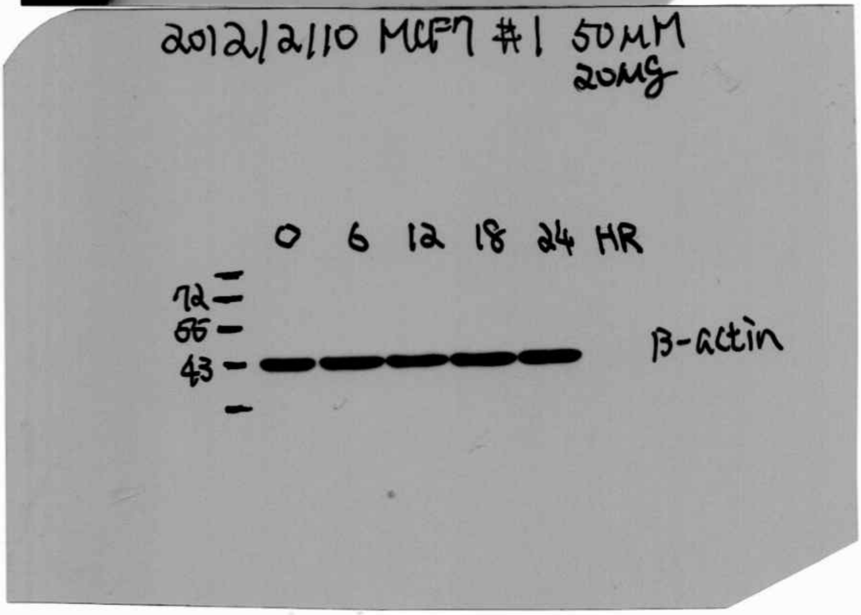

Fig2B beta-actin

Fig2B CDK6

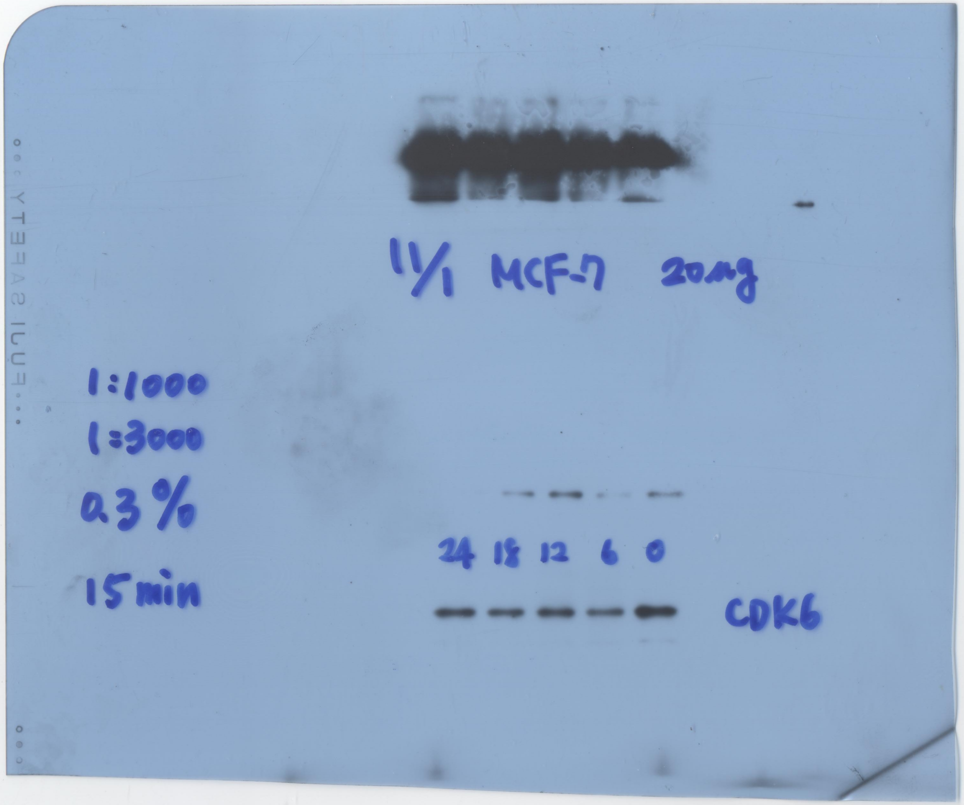

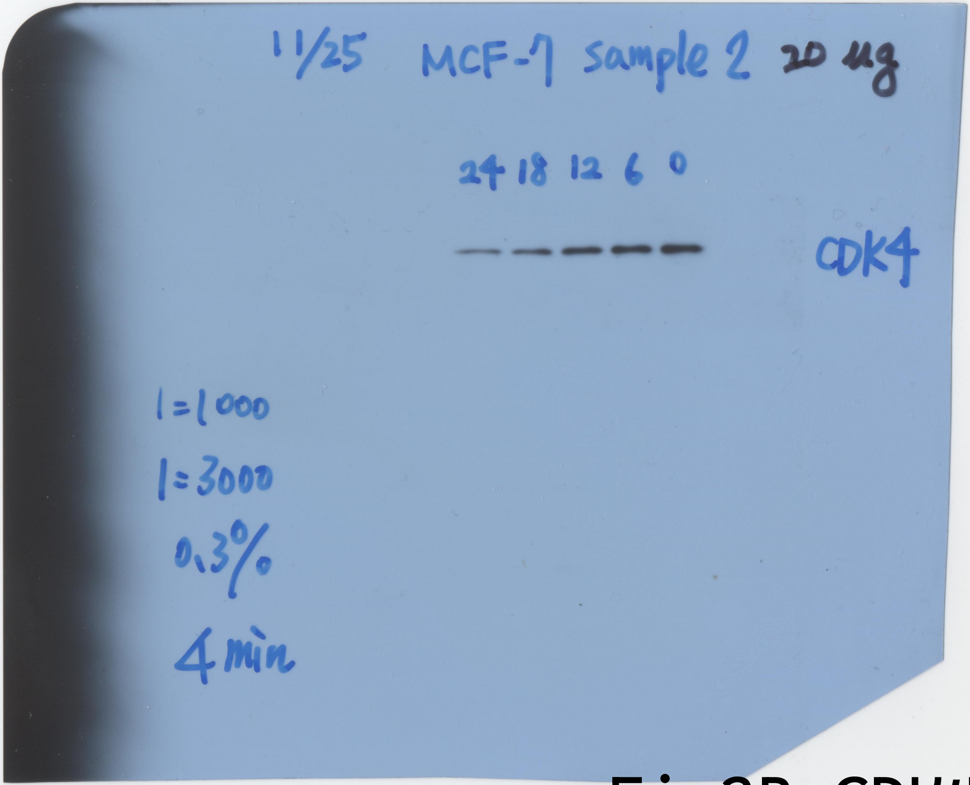

Fig2B CDK4

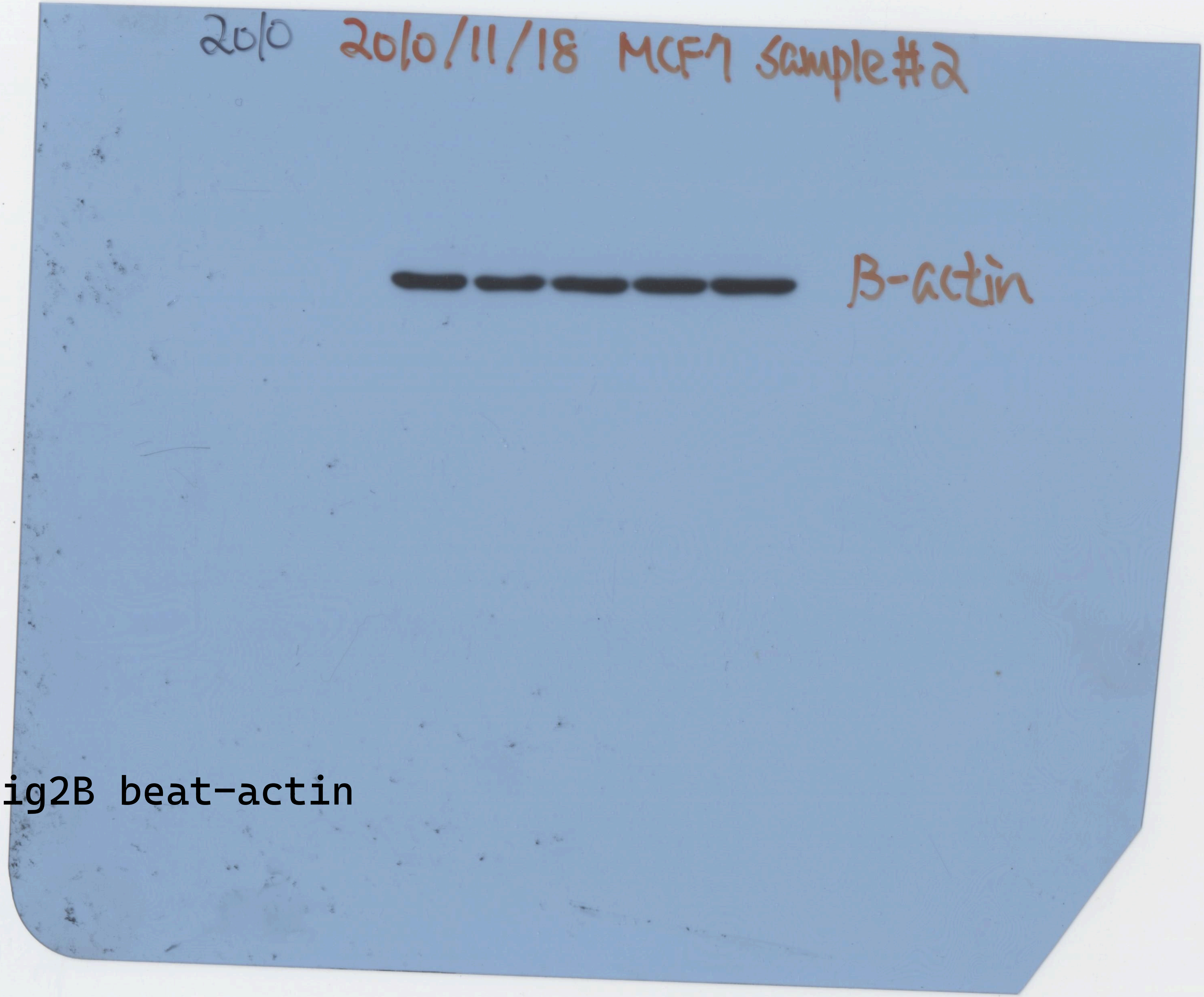

Fig2B beat-actin

Fig2B Cyclin D1

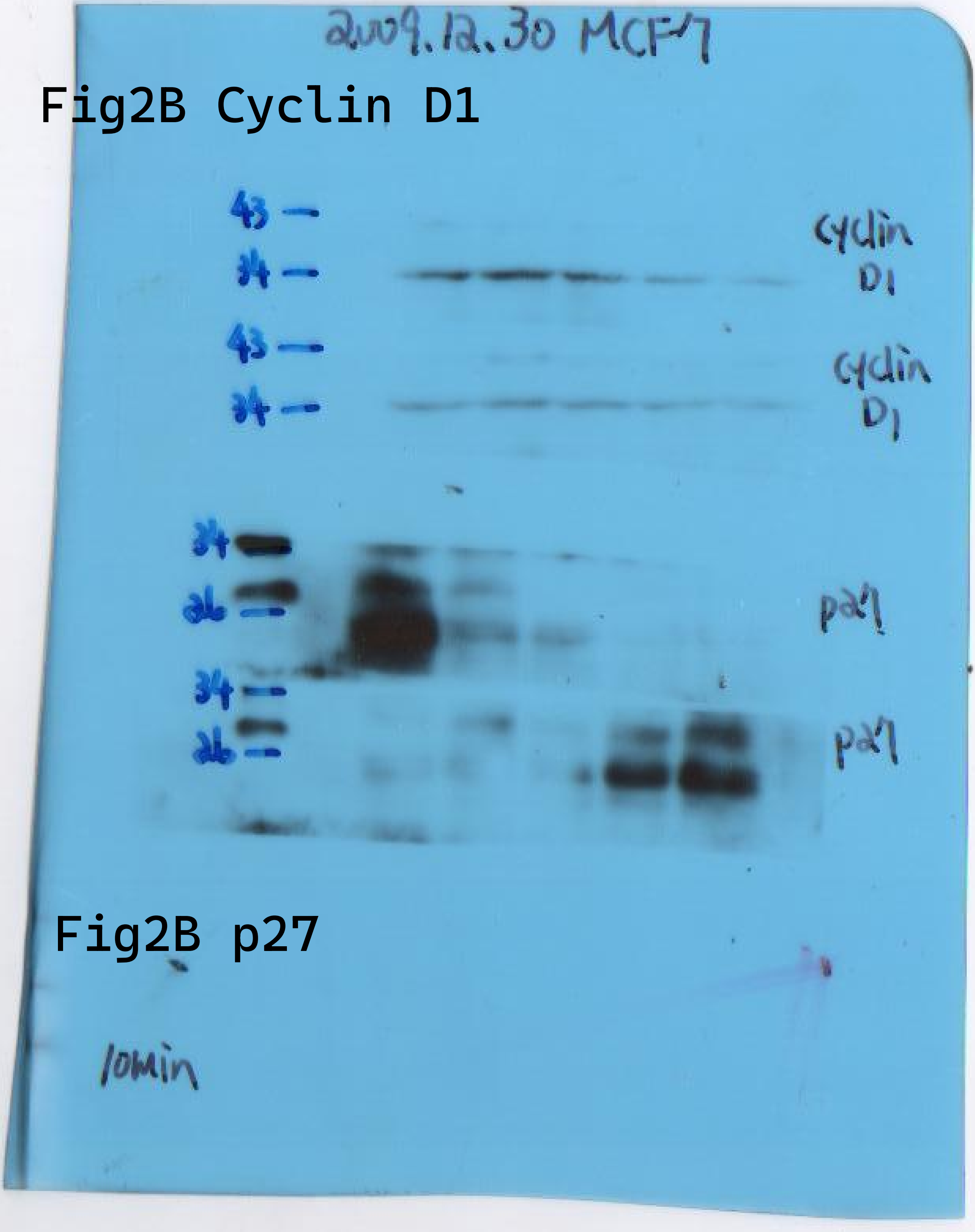

Fig2B p27

Fig2B p21

(supporting data)

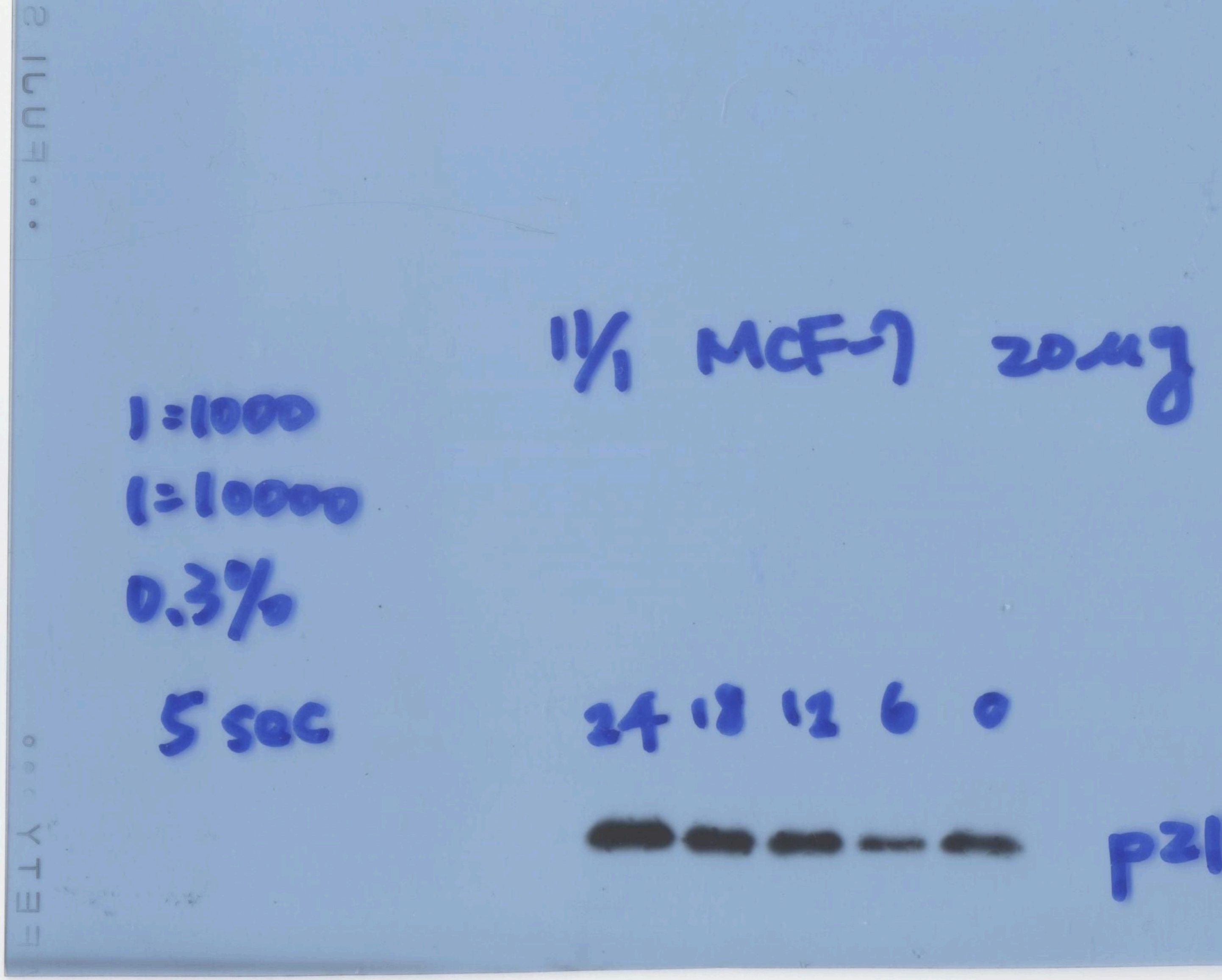

Fig2B p21
(supporting data)

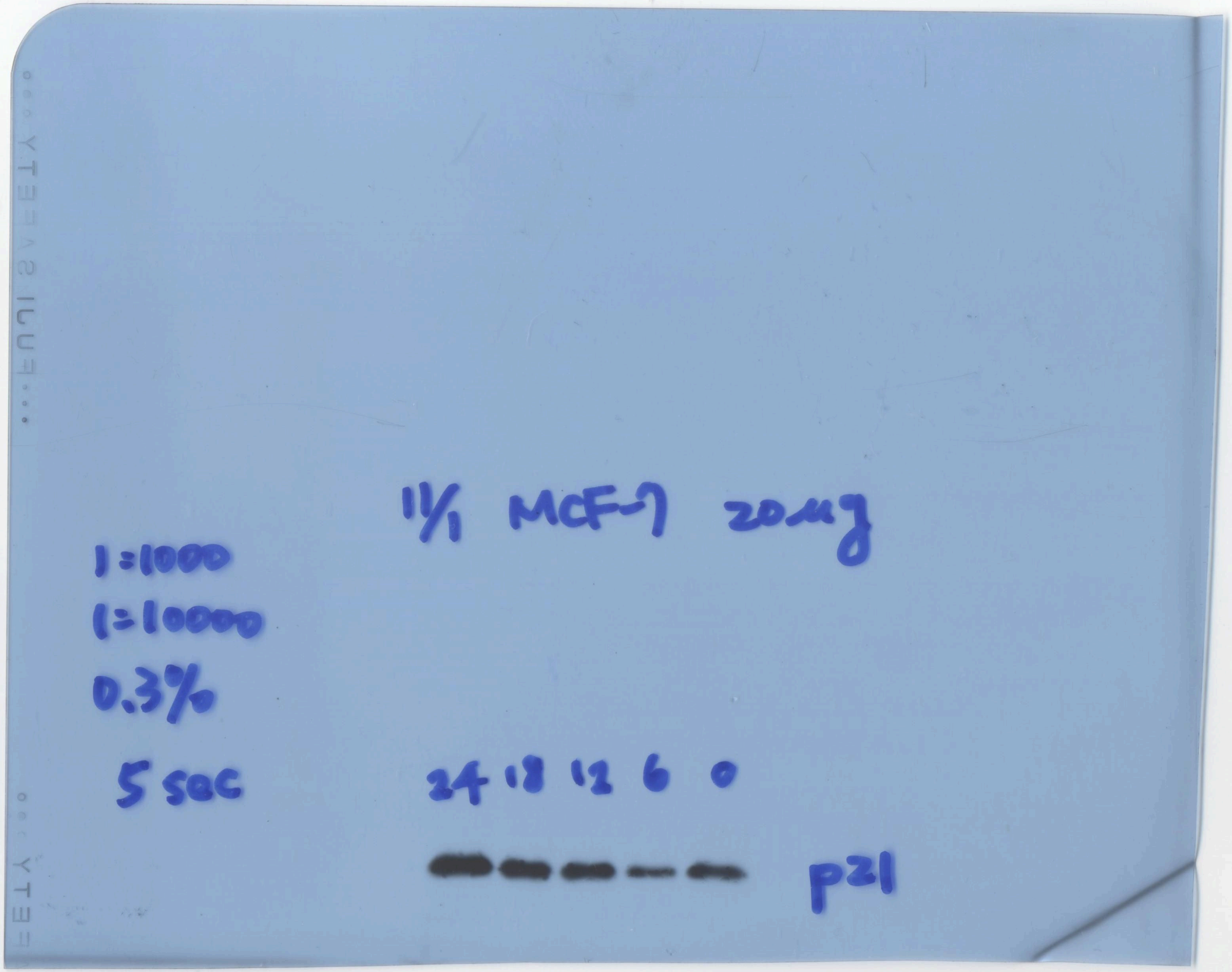

Fig3B Atg5

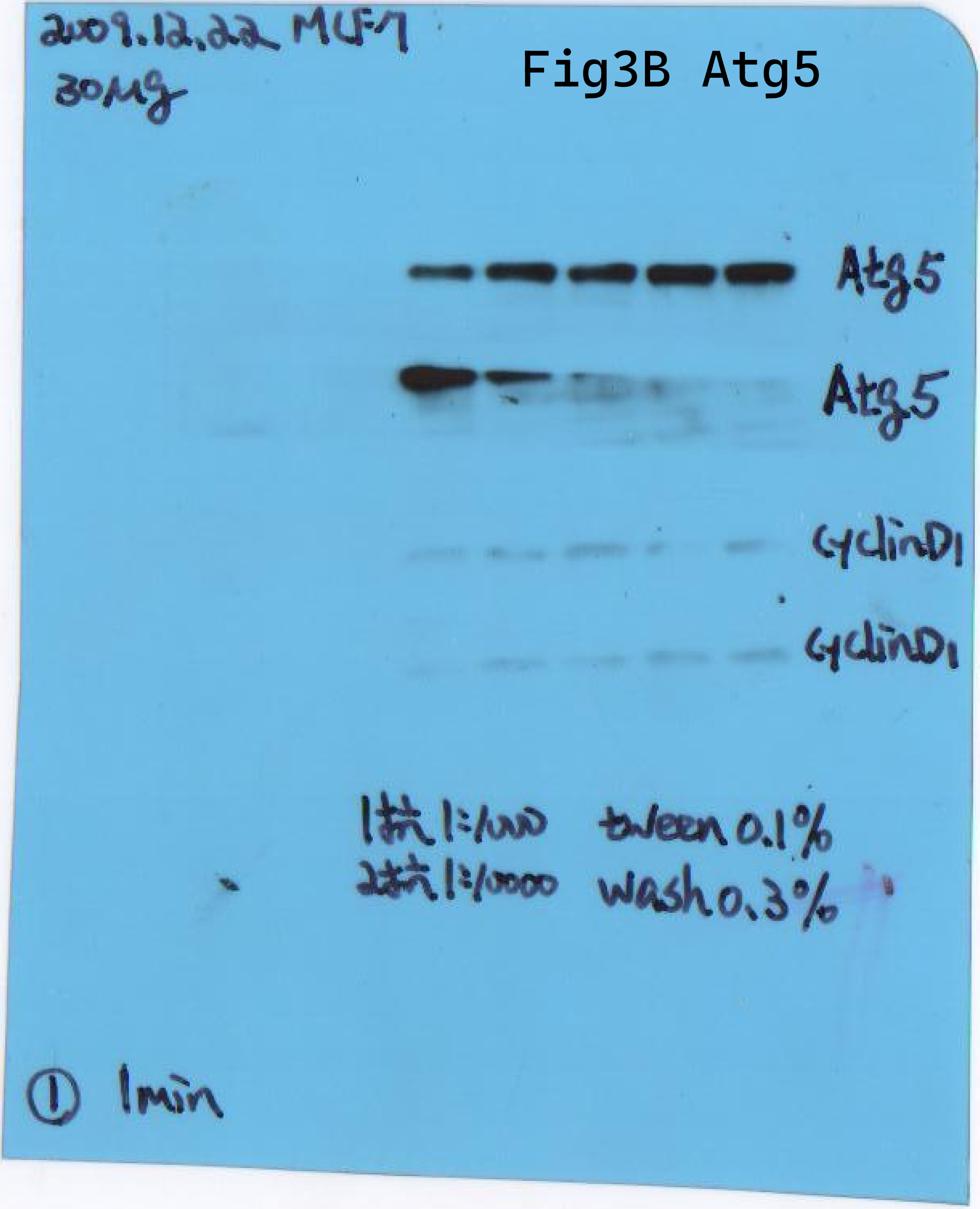
2009.12.22 MCF-7
30μg
Atg5
Atg5
CyclinD1
CyclinD1
1抗 1:1000 tween 0.1%
2抗 1:10000 wash 0.3%
① 1min

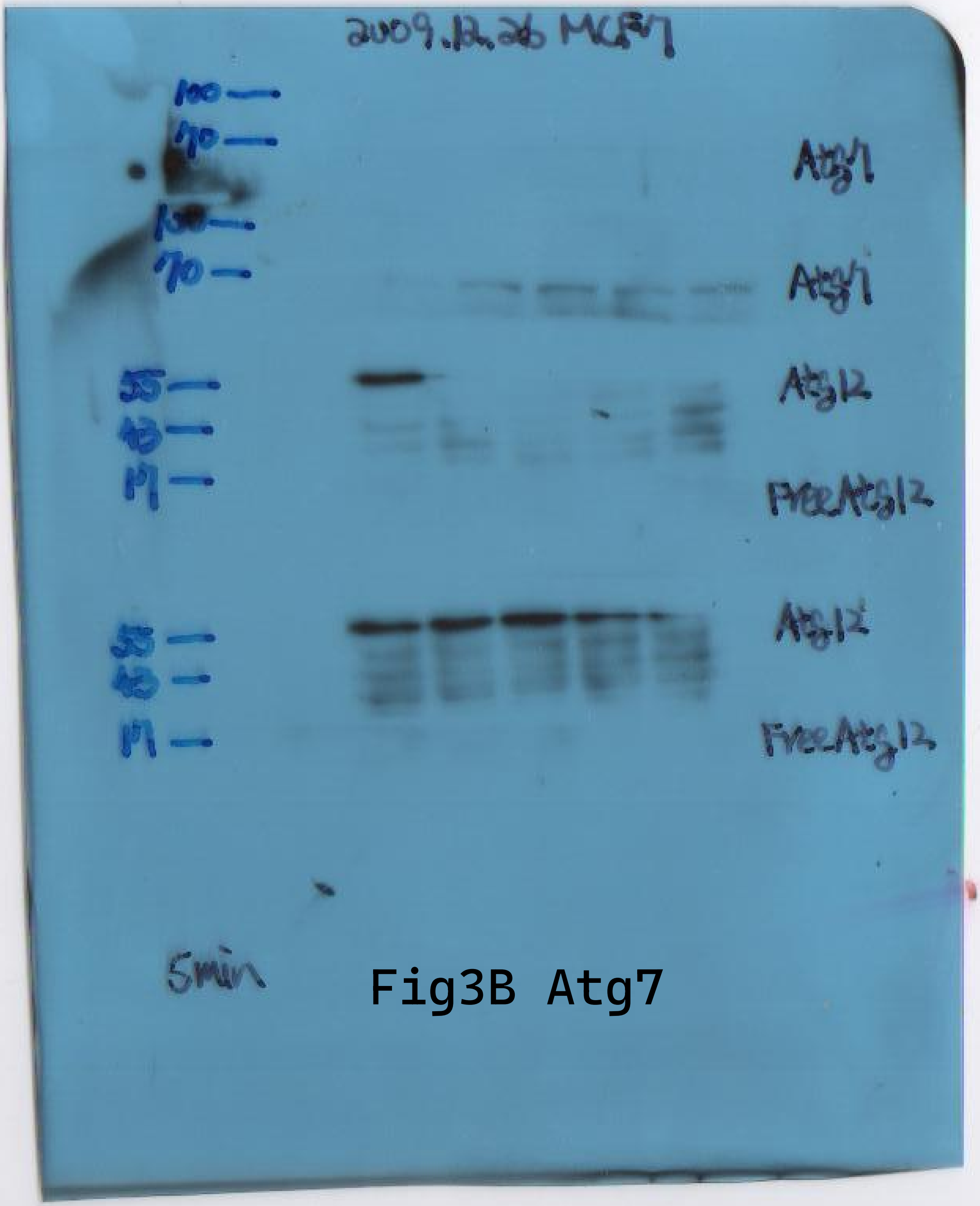
2009.12.26 MCF7
100
70
130
70
55
43
17
55
43
17
Atg7
Atg7
Atg12
Free Atg12
Atg12
Free Atg12
5min

Fig3B Atg7

Fig3B Beclin-1

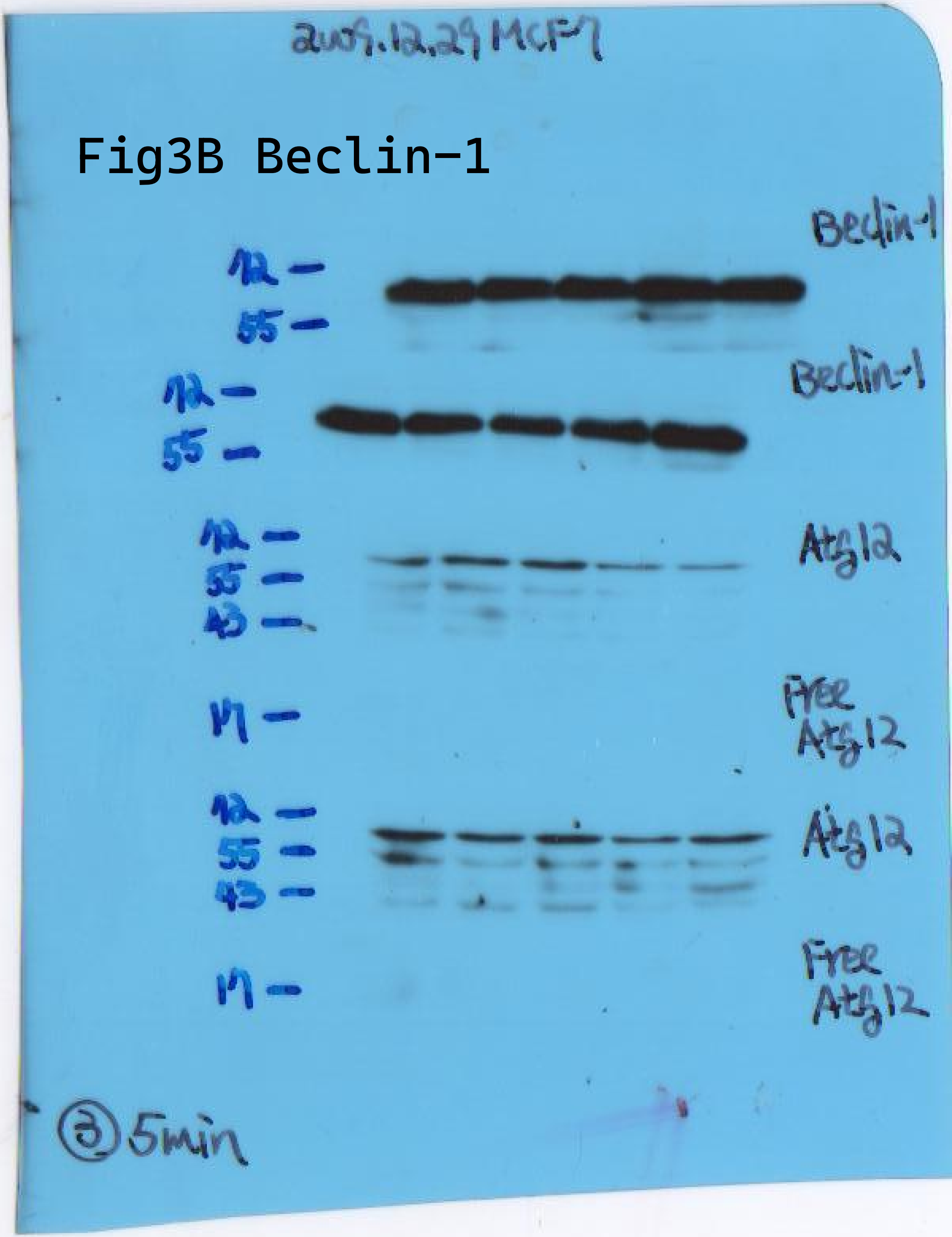

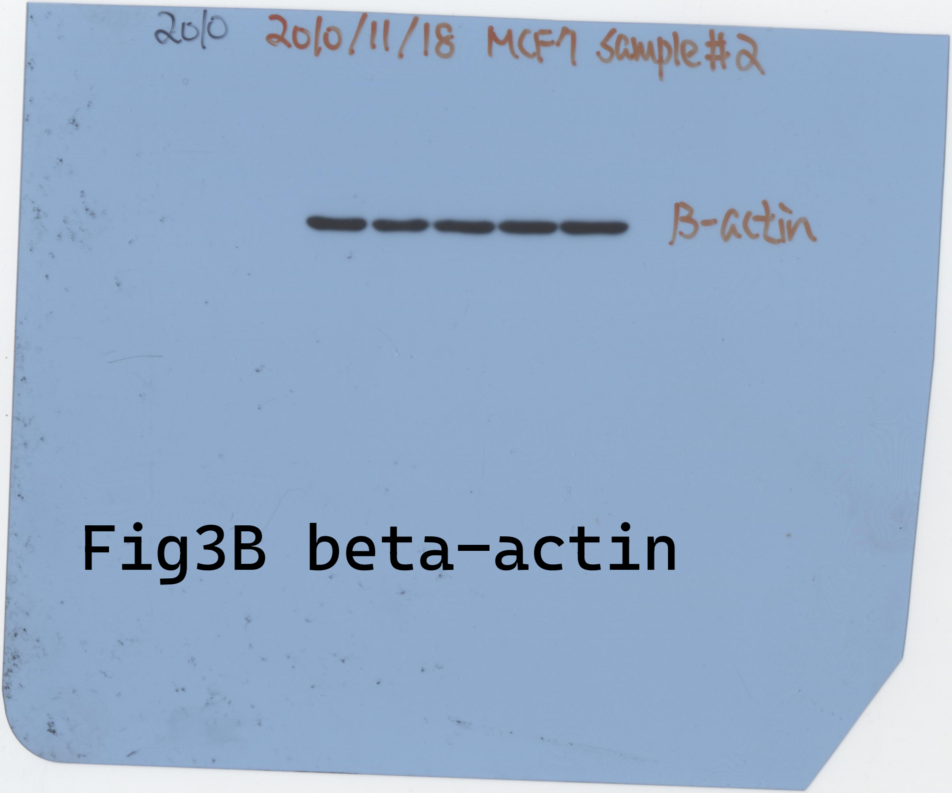

Fig3B beta-actin

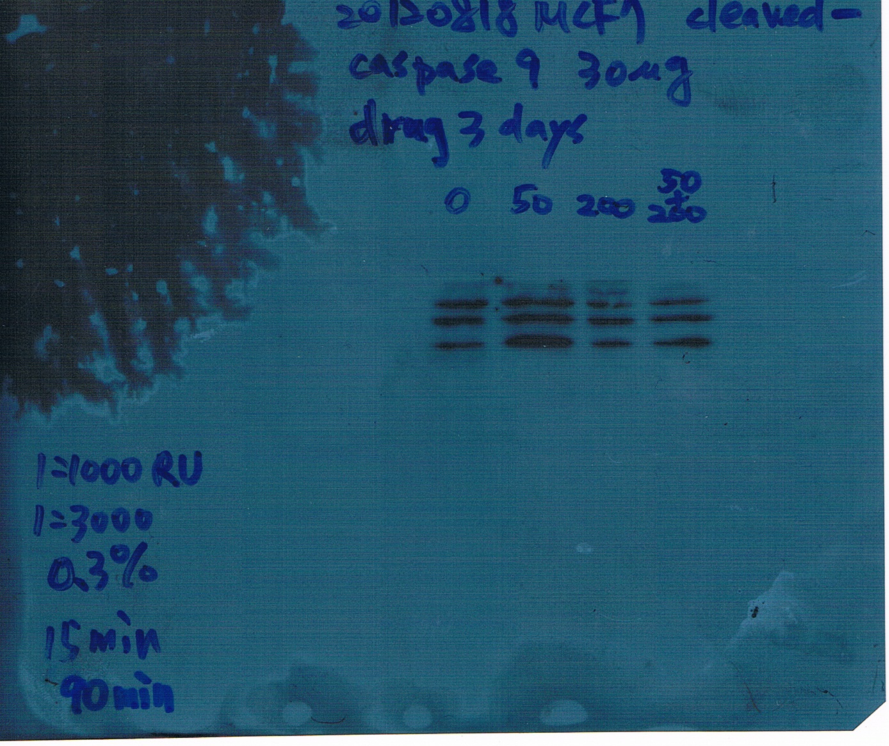

Fig 5C cleaved caspase 9

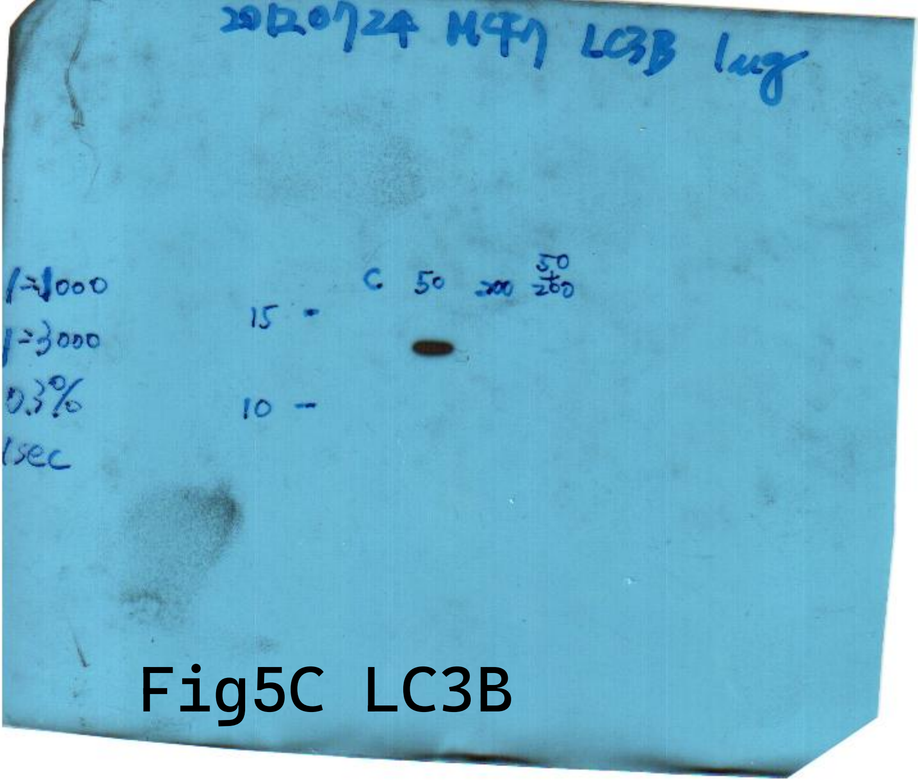
20120724 MCF7 LC3B 1ug
1:1000
1:3000
0.3%
1sec
15
10
C
50
200
50
200
Fig5C LC3B

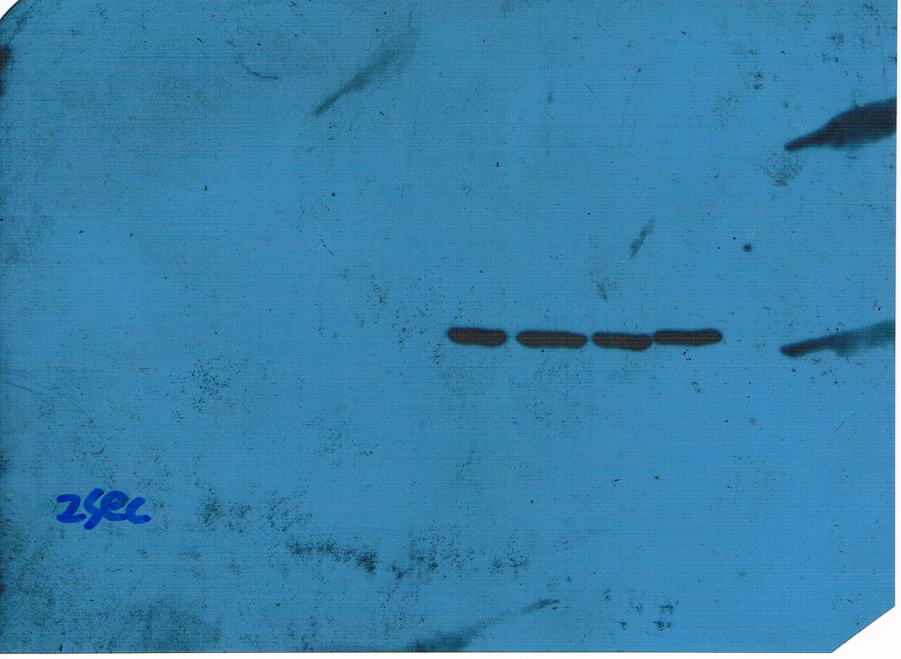

Fig5C beta-
actin

Fig6D cleaved caspase 9

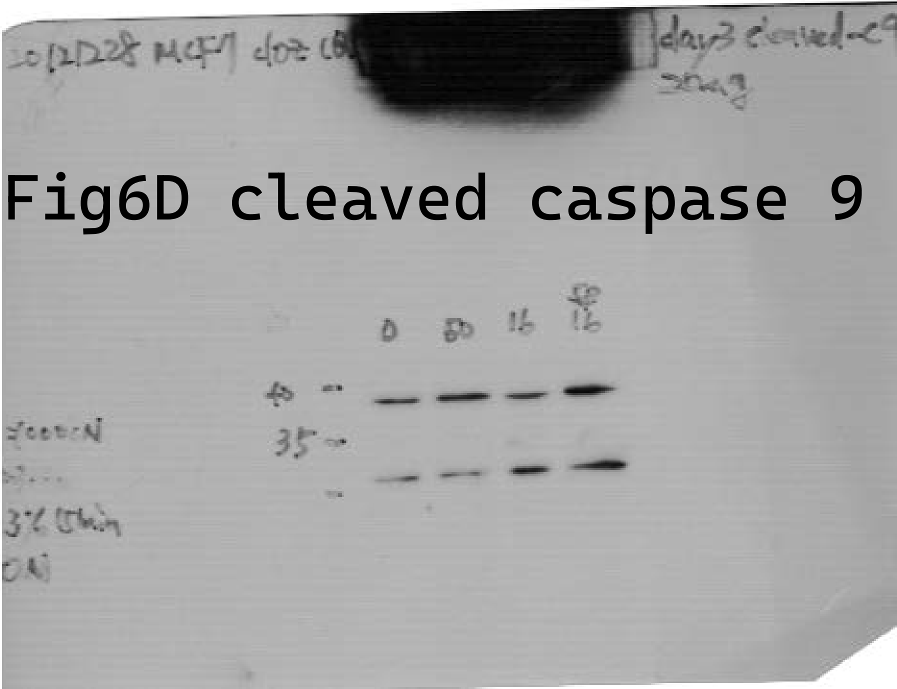

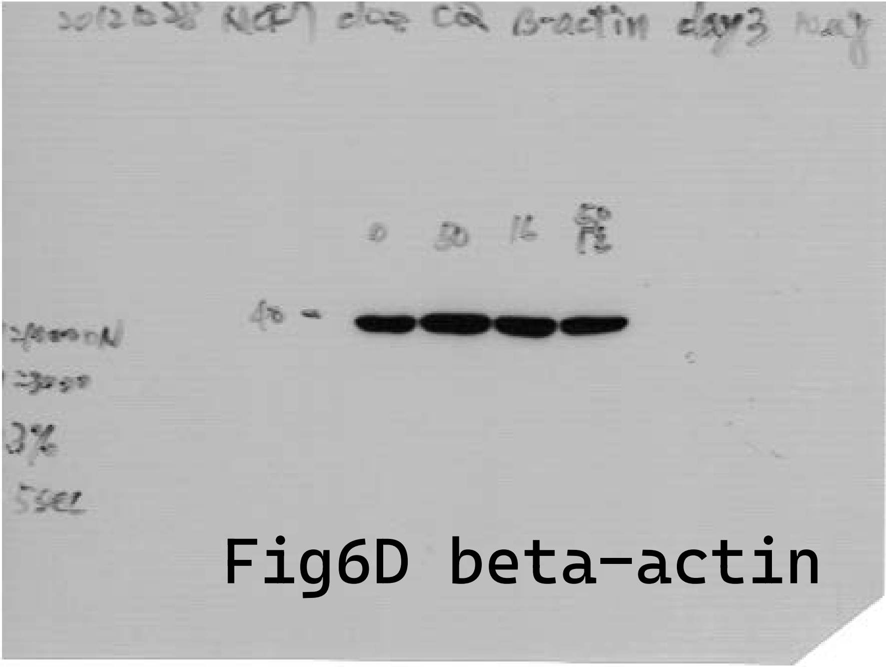

Fig6D beta-actin
